# A cohort study of the effects of older adult care dependence upon household economic functioning, in Peru, Mexico and China

**DOI:** 10.1371/journal.pone.0195567

**Published:** 2018-04-13

**Authors:** Maëlenn M. Guerchet, Mariella Guerra, Yueqin Huang, Peter Lloyd-Sherlock, Ana Luisa Sosa, Richard Uwakwe, Isaac Acosta, Peter Ezeah, Sara Gallardo, Zhaorui Liu, Rosie Mayston, Veronica Montes de Oca, Hong Wang, Martin J. Prince

**Affiliations:** 1 King’s College London, Institute of Psychiatry, Psychology and Neuroscience, Health Service and Population Research Department, London, United Kingdom; 2 Psychogeriatric Unit, National Institute of Mental Health “Honorio Delgado Hideyo Noguchi”, Lima, Peru; 3 Peking University, Institute of Mental Health, Beijing, China; 4 School of Development Studies, University of East Anglia, Norwich, United Kingdom; 5 National Institute of Neurology and Neurosurgery of Mexico, Universidad Nacional Autónoma de México, Mexico City, Mexico; 6 Nnamdi Azikiwe University Teaching Hospital, Nnewi, Anambra State, Nigeria; 7 Department of Sociology/Anthropology, Nnamdi Azikiwe University, Awka, Nigeria; 8 Instituto de la Memoria, Depresión y Enfermedades de Riesgo (IMEDER), Lima, Peru; 9 Instituto de Investigaciones Sociales, Universidad Nacional Autónoma de México, Mexico City, Mexico; Universita degli Studi di Perugia, ITALY

## Abstract

**Background:**

While links between disability and poverty are well established, there have been few longitudinal studies to clarify direction of causality, particularly among older adults in low and middle income countries. We aimed to study the effect of care dependence among older adult residents on the economic functioning of their households, in catchment area survey sites in Peru, Mexico and China.

**Methods:**

Households were classified from the evolution of the needs for care of older residents, over two previous community surveys, as ‘incident care’, ‘chronic care’ or ‘no care’, and followed up three years later to ascertain economic outcomes (household income, consumption, economic strain, satisfaction with economic circumstances, healthcare expenditure and residents giving up work or education to care).

**Results:**

Household income did not differ between household groups. However, income from paid work (Pooled Count Ratio pCR 0.88, 95% CI 0.78–1.00) and government transfers (pCR 0.80, 95% CI 0.69–0.93) were lower in care households. Consumption was 12% lower in chronic care households (pCR 0.88, 95% CI 0.77–0.99). Household healthcare expenditure was higher (pCR 1.55, 95% CI 1.26–1.90), and catastrophic healthcare spending more common (pRR 1.64, 95% CI 1.64–2.22) in care households.

**Conclusions:**

While endogeneity cannot be confidently excluded as an explanation for the findings, this study indicates that older people’s needs for care have a discernable impact on household economics, controlling for baseline indicators of long-term economic status. Although living, typically, in multigenerational family units, older people have not featured prominently in global health and development agendas. Population ageing will rapidly increase the number of households where older people live, and their societal significance. Building sustainable long-term care systems for the future will require some combination of improved income security in old age; incentivisation of informal care through compensation for direct and opportunity costs; and development of community care services to support, and, where necessary, supplement or substitute the central role of informal caregivers.

## Introduction

The inverse correlation between disability and economic status is well established. In the 49 country World Health Survey (over 200,000 adults aged 18 years and older) disability was more prevalent in the poorest than in the richest wealth quintiles in all countries, with a statistically significant gradient in all but six countries [1]. While disability was more prevalent in lower income countries, the inequality gradient was steeper in high- and upper middle-income countries. However, there has been relatively little research on the links between health, disability and poverty in low and middle income countries (LMIC), particularly the impact of care dependence among older adults. In 2011 a critical review included only 27 relevant publications, just 14 focusing on associations between disability and poverty, four among older adults [2]. We updated this review from 2011 to present, not limited to LMIC, but restricted to studies focusing on older adults, using the search terms (poverty AND (health OR disability OR dependen*) AND (old* or age*)). Cross-sectional surveys from Latin America [3–5] and Asia [6,7] demonstrate that older people with disabilities are more commonly to be found living under adverse socioeconomic conditions, usually quantified in terms of current household assets. These associations were not confirmed in two studies from Nigeria [8,9]. While direction of causality cannot be determined from cross-sectional studies it is clear that the focus of interest for much of this research was whether adverse economic conditions lead to poor health and disability. Evidence from HIC suggest that unhealthy ageing trajectories may be determined, partly, by early life socioeconomic disadvantage, or its cumulative effects across the life course [10,11]. Tentative evidence based upon retrospective recall of early life exposures supports similar conclusions from studies in Latin America [3,4].

It is also possible that the onset of chronic ill health, disability and needs for care in an older person impoverishes their household. Plausible mechanisms include work incapacity, family carers cutting back on paid work, increased costs of living, and the costs of health and formal paid care. Several strands of evidence from HIC support this conclusion. In the USA, pre-retirement disability shocks among those aged 51–56 were associated with declining incomes and increased poverty rates over the subsequent eight years [12]. Public and private benefits replaced less than half of the income loss. Analysis of Medicare recipient data indicated that those who had had a hip fracture, compared with a non-exposed cohort were more than twice as likely, over the next year to become dependent on Medicaid or eligible for low-income subsidies [13]. An analysis of data from the USA Survey of Income and Program Participation examined the effect of veteran and disability status on poverty and material hardship among households with an older adult resident. For non-veteran households the presence of an older adult with disability was associated with poverty and economic hardship [14]. While veteran status mitigated against poverty, disabled veterans still experienced high rates of economic hardship (accessing medical care, paying bills and having sufficient food). In the national Australian Survey of Disability, Ageing and Carers, those aged 45–64 who had retired early due to ill health were twice as likely to be in income poverty than those retiring for other reasons, with increased risks of income poverty extending to other family members [15]. Economists have indirectly modelled the costs of disability among older residents, at household level. In Ireland, these were estimated by comparing the standard of living of households with and without older members living with disability, at a given income, controlling for other covariates [16]. The additional economic cost amounted to one-third of household income, varying by disability severity, and was proportionately greater in smaller households. In the UK, among pensioner households, additional cost estimates varied by household composition, 43–50% for single pensioner households, 16% for pensioner couples with one disabled, and 20–50% where both were disabled [17]. The few estimates of extra costs from LMIC are somewhat lower [18], for example, in a similar modelling exercise using survey data from Vietnam the extra cost of living with disability amounted to 9% of annual household income, or US$217 [19]. Estimated costs were considerably higher among older (US$667) than younger people (US$187). These ‘standard of living’ estimates capture neither the direct costs associated with disability, such as health and social care, nor the opportunity costs, such as potential foregone earnings.

Our own 10/66 Dementia Research Group (10/66 DRG) population-based surveys in urban and rural catchment area sites in Latin America, India and China showed a consistent tendency for dependence (needs for care) to be inversely associated with educational level [20]. Dementia was the leading contributor to disability and needs for care among older people [20,21]. Among carers of older people with dementia, cutting back or giving up work to care was common [22], and strongly associated with role strain [23]. More detailed studies of the correlates of care dependence in the Dominican Republic and rural Nigeria further attested to the risk of economic vulnerability [5,9]. Dependent older people were less likely than others to have paid work, and, in Dominican Republic less likely to be in receipt of a pension. In Nigeria they were less likely and in Dominican Republic no more likely than others to benefit from financial support from their family. The 10/66 DRG mixed methods INDEP study is designed to provide a more detailed picture of social and economic consequences of chronic and incident needs for care in older age in selected LMIC [24]. There are three key elements of the quantitative part of the study, conducted in rural and urban sites in Peru, Mexico and China. First, we study social and economic impact at the household level, classifying households according to the needs for care of older residents at the time of the baseline and incidence wave surveys, and introducing a longitudinal perspective by following up the selected households three years after the incidence wave. Second, we assess economic impact more directly than in previous studies, through household consumption and income, as well as assets, indicators of economic strain, and the direct costs of health and social care. At the incidence wave survey, assets were similarly distributed between households selected for ‘care’ and ‘no care’ groups [24]. The accompanying qualitative case studies enabled us to explore mechanisms underlying any observed associations between care dependence and household impoverishment, including factors that support economic resilience [24,25].

## Materials and methods

### Ethical considerations

The INDEP study protocol has been approved by King’s College London Research Ethics Committee and relevant local authorities in each study site: Memory, Depression Institute and Risk Diseases (IMEDER) Ethics Committee in Peru; Instituto Nacional de Neurología y Neurocirugía Ethics Committee in Mexico; Medical Ethics Committee of Peking University the Sixth Hospital (Institute of Mental Health) in China; Nnamdi Azikiwe University Teaching Hospital Nnewi Anambra State Ethics Committee in Nigeria. Participation was on the basis of informed, signed consent. According to our previous survey data, up to half of the older people in the incident care households and two thirds of those in the chronic care households were affected by dementia. We used an approach similar to that used previously in 10/66 studies: if the older person lacked capacity to consent, the next of kin was asked to provide signed assent. Participation was subject to the older person not showing signs of distress or dissent when the information sheet was read to them. For each household, the index older person or persons were first approached for consent for an individual and informant interview, and invited to nominate a suitable key informant for the household interview. If they did not consent, the household was excluded.

### Design

A household cohort study, nested within the prevalence (baseline) and incidence waves of the 10/66 DRG surveys in Peru, Mexico and China. Households were selected on the basis of the needs for care of older residents recorded at baseline and incidence waves (with an interval of 3.5 to 5 years), and then followed up three years after the incidence wave interviews.

### Settings and participants

The INDEP study is conducted in 10/66 survey catchment areas in four countries; China, Peru, Mexico and Nigeria [24]. The INDEP quantitative cohort study was completed in urban and rural sites in Peru, Mexico and China. The urban sites in Peru were Lima Cercado and San Miguel in the capital city, Lima (1381 older people sampled for the baseline survey, conducted in 2005; incidence wave, conducted in 2008, with n = 890 reinterviewed) and rural sites were Cerro Azul, Imperial, Nuevo Imperial, Quilmana, San Luis, and San Vicente in Canete coastal province (baseline survey, 2006, n = 552; incidence wave, 2009, n = 421). The urban sites in Mexico comprised six districts in Tlalpan, Mexico City (baseline survey, 2006, n = 1003 in; incidence wave, 2009, n = 749) and the rural sites comprised nine villages in Morelos, a mountainous district 70km from Mexico City (baseline survey, 2006, n = 1000; incidence wave, 2009, n = 713). The urban site in China was Xicheng, close to Tiananmen Square in Beijing City (baseline survey, 2004, n = 1160; incidence wave, 2009, n = 741), while the rural site comprised 14 villages in Daxing, a rural district 40 kilometres away (baseline survey, 2004, n = 1002; incidence wave, 2009, n = 711). The catchment area sites are not nationally representative, nor even necessarily representative of the city or rural region where they are located. Urban areas were selected to be predominately lower socioeconomic status, or mixed neighborhoods, avoiding middle class or professional enclaves [26]. Rural areas were selected to be distant from conurbations, and to include a high proportion of inhabitants with agrarian occupations.

### Sampling

For the INDEP study, we sampled in each site from among those households where one or more older participants (referred to as ‘index older people’ or IOP) had been interviewed at the baseline and incidence waves, categorizing these households as follows.

1. Incident care households (where all IOP were independent at baseline, but in which one or more had become care dependent by the incidence survey).
2. Chronic care households (with one or more care dependent IOP at baseline, who remained care dependent in the incidence survey).
3. No care households (where all IOP were independent at baseline, and remained so at the incidence survey).

All households meeting criteria for incident or chronic care were selected for the INDEP study. In each site, no care households equivalent in number to the sum of incident and chronic care households were selected at random from all those eligible, frequency matched to care households for the age of the oldest resident (in four groups; age 65–69, 70–74, 75–79 and 80+). This approach avoided what would otherwise have been marked differences in the age distributions of older adult residents between care and no care households, age being an important determinant of many health and social outcomes other than care dependence, which might, themselves, be independently associated with the economic outcomes studied.

### Household tracing and redesignation

We envisaged that, when recontacted for the INDEP study in 2012, three years after the mid-point of the incidence wave surveys, there would have been changes in household composition and needs for care [24]. When all IOP who needed care (in incident or chronic care households) had died, the household was redesignated as a ‘care exit’ household, and only the household interview was completed. When all IOP in no care households had died, the household was excluded from the INDEP study. If all surviving IOP with needs for care had moved to another household then the household to which they had moved was redesignated as the household of interest. If two or more had moved to separate households, we followed the IOP with the highest level of needs for care at the incidence wave survey. If all surviving IOP from no care households had moved to another household then the household to which they had moved was redesignated as the household of interest. If two or more had moved to different households, we followed the youngest. Needs for care of all IOPs were reappraised in the INDEP study informant interview. Where needs for care had developed for one or more IOP in no care households, such households were still included as no care households in the main analysis, but were then excluded for the sensitivity analysis (see below).

### Data collection

For each selected household, we aimed to conduct a household interview with a suitable key informant (usually the head of household), brief interviews with each of the surviving IOP, and an interview with an informant for each IOP for an independent perspective on their health and needs for care. All interviews were conducted masked to household group status.

### Measures

A full account of the interviews administered in the INDEP study is provided in our open access protocol paper [24]. Here we summarise measures used for the current analyses. Household income and consumption were not assessed in previous 10/66 surveys. INDEP study assessments were developed from questionnaires used in community research into social pensions, poverty and wellbeing in South Africa and Brazil [27]. We checked with local investigators the relevance and comprehensiveness of questions regarding sources of income and types of expenditure, and adjusted the questions to reflect local systems. The detailed household interview comprises:

1. Household composition and roles—the age, sex, marital, educational and occupational status of all residents.
2. Economic evaluation
  - A household assets index covering household goods and amenities (telephone or mobile phone, stove, electricity supply, television, radio or stereo, refrigerator, sewing machine, bicycle, computer, and motor vehicles).
  - Monthly household income was estimated by enquiring about 20 different sources of income and allocating each to an individual resident, or to the household if not specifiable. Income sources were clustered into five groups; pensions (government social pensions, employer pension or retirement annuity), paid work (full or part-time regular or occasional work, or income from a business, and any employment benefits), income from assets (savings, investments, property rents, lodgers), government transfers (unemployment benefit, child support grants, disability benefits, public work schemes) and private transfers (money from religious organisations, non-governmental organizations (NGOs) or charities, gifts or regular payments from family or others outside of the household). Total monthly household income was calculated by summing after tax income across all sources and all residents. This monthly amount was then equivalised by dividing by the modified Organisation for Economic Cooperation and Development (OECD) equivalence scale (1.0 for the first adult, 0.5 for all other adults, and 0.3 for children) to account for economies of scale, and converted into 2011 international dollars using PPP exchange rates [28].
  - Consumption, 25 items eliciting food consumption (the value or cost of all food consumed at home and outside of the home), household expenses and other personal expenditure, also divided by the OECD equivalence scale. Consistent with convention, health and social care expenses were not included in general consumption, but considered separately. Catastrophic healthcare costs were defined as spending more than 10% of household income in the last three months on health care.
  - Indicators of household financial strain over the last three years. These included; asking for help from friends or relatives, an employer, a religious organisation, or charity; taking a loan; cutting down on food consumption; seeking extra work; running up an account with a shop; applying for a grant; apply for food parcels or vouchers; drawing on savings, selling stocks or shares; any other action to address the financial difficulty. The number of indicators endorsed was grouped into three categories for the analyses; none, one, and two or more.
  - Subjective assessment of overall financial status; How would you rate the financial situation of this household at present? For the purpose of analysis this was grouped into three categories, very good or good, average, and bad or very bad.

### Analyses

1. All analyses were weighted to take account of sampling fractions of care and no care households, and non-response at household level, aiming for generalizability to the incidence phase of the 10/66 surveys in each catchment area site [24,26]. Non-response-adjusted sampling weights were derived by first calculating sampling weights as the inverse of the selection probability for each site, household group and older resident age group. We then calculated response weights as the inverse of the response proportion for each of these groups. The non-response adjusted sampling weight was the product of these two weights. Weighted analyses were conducted by using the Stata ‘pweight’ sample weight sub-command.
2. We summarize, for each site, the distribution of household size, composition, and socioeconomic status (household assets, and occupational status of the IOP), as assessed at the incidence wave of the 10/66 survey, and three years later in the nested INDEP study, and their crude association with household care status (no care households vs care households [incident or chronic]).
3. The general approach for testing the main hypotheses was to compare, as exposures, no care households with each of the other three care categories (incident care, chronic care and care exit households). We also compared ‘current care’ households (incident and chronic care households combined) with no care households, omitting care exit households. The preselected outcomes were total household equivalised income, total household equivalised consumption, economic strain, satisfaction with economic circumstances, healthcare expenditure, catastrophic healthcare expenditure, and co-residents giving up work or education to provide care for an older adult. Secondary analyses looked at sub-categories of income (from paid work, pensions, private and government transfers, and assets) and consumption (food consumption). Regression models were selected depending on the distributional characteristics of outcome data. Negative binomial regression (generating count ratios) was used for the main income and consumption outcomes, which were over-dispersed (as established from the Alpha dispersion coefficient, and likelihood ratio tests). Zero-inflated negative binomial regression (generating count ratios) was used for household income from paid work, pensions, transfers and assets, and for household healthcare expenditure, which were also characterized by excess zeros (as established with a Vuong test). Ordinal regression (odds ratios across ordinal categories) was used for economic strain and dissatisfaction with economic circumstances. Poisson regression (prevalence ratios) was used for the two dichotomous outcomes, catastrophic healthcare expenditure and giving up work or education to care for an older person. All models were adjusted for the potential confounding effects of household composition and economic status (household assets, and occupational status of the IOP) at the time of the incidence wave. The effects of household care status on income from paid work (non-equivalised) were further controlled for the number of working age adult residents; on pension income (non-equivalised) for the number of older persons; and on healthcare expenditure and catastrophic healthcare expenditure for the number of child and number of adult residents (since to different extents in the different health systems the numbers of residents in these age groups might be an important determinant of household-level demand for healthcare, access to healthcare, and ensuing out-of-pocket expenditure).
4. All models were fitted separately for each site and we then used a fixed effects meta-analysis to combine them, hence maximizing power and precision. Higgins I^2^ quantifies the proportion of between-site variability accounted for by heterogeneity, as opposed to sampling error; up to 40% heterogeneity is conventionally considered negligible, while up to 60% may reflect moderate heterogeneity [29].

#### Sensitivity analysis

We re-estimated the effects of household care status on the main outcomes, excluding households subject to household changes, and no care households where IOPs had developed needs for care.

## Results

### Sample characteristics

One thousand three hundred and fifty-four households were selected for the INDEP nested cohort study, on the basis of needs for care for older adults observed in the baseline and incidence wave 10/66 surveys (Table 1). Of these, 493 were incident care households, and 189 chronic care households; 672 age-matched no care households were selected, slightly fewer than the planned one no care household per care household since insufficient age-matched no care households were available in urban Mexico and urban China. Consistent with the study protocol, we then reclassified the households based upon their composition when revisited for the INDEP survey. Sixty-eight (10%) of the no care households were redesignated as ‘lost’ since all older residents had died, and these were excluded from the INDEP survey. 199 (40%) of the incident care households and 89 (47%) of the chronic care households were redesignated as care exit households, since all of the older persons requiring care were found to have died. Therefore, the final redesignated household classification for the main analysis comprised 1286 eligible households; 604 control households, 294 incident care households, 100 chronic care households and 288 care exit households.

**Table 1:** Original household designation (at the time of selection) and redesignation (upon tracing for INDEP survey).

Original household designation	Peru urban	Peru rural	Mexico urban	Mexico rural	China urban	China rural	All sites
No care	138	49	123	112	168	82	672
Incident care	87	38	84	87	124	73	493
Chronic care	51	11	37	25	56	9	189
Total	276	98	244	224	348	164	1354
Redesignation process^1^	Peru urban	Peru rural	Mexico urban	Mexico rural	China urban	China rural	All sites
No care> No care lost	21 (15%)	4 (8%)	17 (14%)	13 (12%)	10 (6%)	3 (4%)	68 (10%)
Incident care> care exit	36 (41%)	10 (26%)	35 (42%)	34 (39%)	51 (41%)	33 (45%)	199 (40%)
Chronic care> care exit	23 (45%)	4 (36%)	18 (49%)	12 (48%)	25 (45%)	7 (78%)	89 (47%)
Redesignated household categories	Peru urban	Peru rural	Mexico urban	Mexico rural	China urban	China rural	All sites
No care	117	45	106	99	158	79	604
Incident care	51	28	49	53	73	40	294
Chronic care	28	7	19	13	31	2	100
Care exit	59	14	53	46	76	40	288
Total	255	94	227	211	338	161	1286

^1^. number, and percentage of all those in the original designation

Household interviews were successfully completed for 872 of the 1286 eligible households (68%), with overall response rates varying from 52% (China urban) to 89% (China rural) (Table 2). Household interviews were completed on 424 no care households (70% of those eligible), 227 incident care households (77%), 67 chronic care households (67%) and 154 care exit households (54%). Where household interviews were completed, we were generally also successful in interviewing surviving older residents. All those eligible were interviewed in 93% of households with surviving older residents, and at least some eligible older residents were interviewed in a further 6% of such households.

**Table 2:** Response proportions for household interview and individual older person interview^1^, at household level, by site.

Site	Interview	Incident care	Chronic care	Care exit	No care	All groups
China urban	Household	49/73 (67%)	15/31 (48%)	21/76 (28%)	91/158 (58%)	176/338 (52%)
	Individual	All 48/49 None 1/49	All 15/15	Not required	All 89/91 Some 1/91 None 1/91	All 152/155 Some 1/155 None 2/155
China rural	Household	40/40 (100%)	2/2 (100%)	40/40 (100%)	62/79 (79%)	144/161 (89%)
	Individual	All 39/40 Some 1/40	All 2/2	Not required	All 52/62 Some 10/62	All 93/104 Some 11/104
Peru urban	Household	31/51 (61%)	15/28 (54%)	22/59 (37%)	72/117 (62%)	140/255 (55%)
	Individual	All 27/31 Some 3/31 None 1/31	All 12/15 Some 3/15	Not required	All 66/72 Some 6/72	All 105/118 Some 12/118 None 1/31
Peru rural	Household	17/28 (61%)	5/7 (71%)	6/14 (43%)	28/45 (62%)	56/94 (60%)
	Individual	All 17/17	All 5/5	Not required	All 27/28 Some 1/28	All 49/50 Some 1/50
Mexico urban	Household	44/49 (90%)	18/19 (95%)	36/53 (68%)	91/106 (86%)	189/227 (83%)
	Individual	All 41/44 Some 3/44	All 15/18 Some 3/18	Not required	All 85/91 Some 6/91	All 141/153 Some 12/153
Mexico rural	Household	46/53 (87%)	12/13 (92%)	29/46 (63%)	80/99 (81%)	167/211 (79%)
	Individual	All 44/46 Some 2/46	All 11/12 Some 1/12	Not required	All 76/80 Some 3/80 None 1/80	All 131/138 Some 6/138 None 1/138
All sites	Household	227/294 (77%)	67/100 (67%)	154/288 (54%)	424/604 (70%)	872/1286 (68%)
	Individual	All 216/227 Some 9/227 None 2/227	All 60/67 Some 7/67	Not required	All 395/424 Some 27/424 None 2/424	All 671/718 Some 43/718 None 4/718

^1^. Profile of individual interviews at household level, that is the number of households at which all eligible index older people (all), some eligibles (some) or none of those eligible (none) were interviewed. An IOP was eligible for interview if they were alive and still resident at the household.

The weighted sociodemographic characteristics of the 872 households with completed household interviews are summarized in Table 3. In all sites the mode was for older people to live in multigenerational households with younger adults, and, often, children under the age of 16. The urban China site stood out as having smaller households, a higher proportion of households where older people lived without younger adults (39.2%), and a very low proportion of households with co-resident children (8.3%). The baseline socioeconomic status of households tended to be higher in urban than rural sites, although China rural households were relatively asset rich despite low levels of occupational attainment. Of these characteristics, only household living arrangements were associated with household care status; specifically, older people living alone (9.4% of all households) were under-represented in incident and chronic care groups. Household changes occurred when index older people were followed from the household originally selected to another location to which they had moved since the 10/66 survey. This affected 89 households (10.2%). Care households were marginally more likely to have been subject to household changes, but this was a non-significant trend. Overall, there was very little change in household size from baseline. Neither change in household size, nor assets at follow-up differed by original household care status.

**Table 3:** Sociodemographic characteristics of households completing household interview at time of selection and at INDEP interview^1^, and associations with household care status (weighted analysis^2^).

	Peru urban	Peru rural	Mexico urban	Mexico rural	China urban	China rural	All sites	Association (PR) with household care status (incident and chronic care vs no care)
Number of households (weighted number)	140 (705)	56 (371)	189 (620)	167 (610)	176 (508)	144 (587)	872 (3401)	
At household selection								
Mean number of residents (SD)	4.4 (2.1)	4.0 (2.4)	4.0 (2.8)	3.4 (1.9)	2.8 (1.2)	3.9 (1.7)	3.7 (1.7)	1.04 (0.99–1.08)
Co-resident children aged <16 years (%)	52.1	41.8	34.2	27.0	8.3	22.8	31.6	1.07 (0.87–1.31)
Index older person’s (IOP) living arrangements								
Alone (%)	8.0	14.6	14.7	8.0	5.5	7.2	9.4	1 (ref)
With spouse only (%)	10.5	4.9	6.6	12.8	30.5	21.3	14.4	1.73 (1.06–2.84)
With adult children +/- others (%)	58.4	63.3	63.9	65.6	48.8	59.3	60.0	1.70 (1.08–2.68)
Other arrangement (%)	23.2	17.3	17.3	13.6	15.2	12.3	16.2	1.78 (1.10–2.90)
Mean assets (SD)^3^	6.2 (0.5)	4.9 (1.0)	6.0 (1.0)	4.0 (1.8)	5.4 (0.6)	5.5 (1.3)	5.4 (1.4)	0.99 (0.93–1.06)
Highest occupational status among IOPs (skilled or manual labourer %)	27.9	91.3	60.4	91.3	42.4	96.1	66.1	1.04 (0.96–1.13)^4^
At INDEP interview								
Household change (%)	1.8	2.2	2.4	13.7	5.2	35.1	8.9	1.12 (0.83–1.52)
Mean number of residents	4.4 (2.4)	4.3 (2.4)	3.3 (1.9)	3.8 (2.3)	2.6 (1.3)	4.1 (1.6)	3.7 (2.1)	1.01 (0.97–1.06)
Mean change in number of residents from baseline	-0.1 (2.0)	+0.3 (1.7)	-0.7 (3.0)	+0.5 (2.8)	-0.3 (1.1)	+0.3 (1.8)	0.0 (2.3)	0.98 (0.94–1.02)
Mean assets (SD)^3^	9.1 (1.3)	7.2 (2.5)	8.2 (1.4)	6.4 (1.8)	8.4 (1.5)	8.6 (1.9)	8.0 (2.0)	0.99 (0.94–1.04)

^1^. Households were selected from the incidence wave of the 10/66 survey, and data on household characteristics were collected at that time. Recontacting for INDEP interviews was carried out three years later.

^2^. Weighted for sampling fraction, and response.

^3^. An extended assets scale was used for the INDEP survey, and 10/66 survey and INDEP survey assets data are therefore not directly comparable.

^4^. Per occupational status level.

### Tests of the main hypotheses

While there was no evidence that total household income differed between care and no care household groups, there was a trend for income from paid work to be lower in incident and chronic care households, with a statistically significant pooled effect for the combined current care group (CR 0.88, 95% CI 0.78–1.00, I^2^ = 44.5%) (Table 4). Income from government transfers was also lower, for both incident and chronic care households compared with no care households. Income from pensions was similar among all groups with a trend towards higher levels in care households in some sites. Income from external sources (private transfers) was also similar among sites, but with a trend towards lower levels in care exit households (CR 0.80, 95% CI 0.61–1.05, I^2^ = 0.0%). Income from assets was markedly higher for care households in urban Peru (CR 2.34, 95% CI 1.34–4.08) and urban China (CR 11.1, 95% CI 3.7–33.2), but somewhat lower in rural China (CR 0.51, 95% CI 0.25–1.06)–this source of income was too rare to estimate effects in rural Peru and rural Mexico, and heterogeneity among the other sites did not support meta-analysis.

**Table 4:** Associations between redesignated household status (no care versus incident care, chronic care and care exit households) and main household income and its sub-categories (income from paid work, pensions, private and government transfers).

**Equivalised**^2^ **household income** Negative binomial regression—adjusted^3^ count ratios	Site/ Country	Current care^1^ n = 292	Incident care^1^ n = 225	Chronic care^1^ n = 67	Care exit^1^ n = 156
	Peru urban	1.01 (0.86–1.18)	0.99 (0.83–1.19)	1.03 (0.82–1.30)	1.01 (0.82–1.23)
	Peru rural	0.84 (0.58–1.22)	0.83 (0.55–1.25)	1.05 (0.52–2.13)	1.00 (0.55–1.83)
	Mexico urban	0.99 (0.81–1.21)	1.03 (0.83–1.30)	0.84 (0.61–1.15)	1.04 (0.80–1.34)
	Mexico rural	0.88 (0.68–1.15)	0.92 (0.68–1.23)	0.84 (0.51–1.38)	1.09 (0.76–1.56)
	China urban	**1.45 (1.07–1.96)**	**1.61 (1.17–2.21)**	1.03 (0.64–1.66)	**0.63 (0.42–0.94)**
	China rural	0.78 (0.49–1.23)	0.79 (0.49–1.29)	**0.19 (0.04–0.99)**	**1.67 (1.04–2.67)**
	Pooled CR	1.00 (0.91–1.10)	1.02 (0.92–1.14)	0.94 (0.81–1.11)	1.02 (0.89–1.16)
	I squared	42.5%	52.5%	6.3%	49.3%
**Household income from paid work (controlling also for number of adults)** Zero inflated negative binomial regression—adjusted^3^ count ratios	Site/ Country	Current care	Incident care	Chronic care	Care exit
	Peru urban	0.82 (0.66–1.03)	0.87 (0.67–1.13)	0.79 (0.57–1.10)	1.01 (0.77–1.33)
	Peru rural	1.06 (0.72–1.55)	1.14 (0.40–2.01)	0.90 (0.40–2.01)	1.64 (0.78–3.44)
	Mexico urban	1.17 (0.83–1.67)	1.36 (0.93–1.99)	0.92 (0.60–1.40)	1.10 (0.73–1.63)
	Mexico rural	1.19 (0.77–1.84)	1.34 (0.82–2.18)	1.05 (0.46–2.42)	1.33 (0.84–2.10)
	China urban	**0.63 (0.42–0.94)**	**0.56 (0.35–0.90)**	1.75 (0.58–5.26)	0.34 (0.16–0.73)
	China rural	0.79 (0.61–1.02)	**0.78 (0.61–0.99)**	1.12 (0.40–3.13)	0.99 (0.78–1.27)
	Pooled CR	**0.88 (0.78–1.00)**	0.90 (0.78–1.04)	0.90 (0.71–1.12)	1.02 (0.88–1.19)
	I squared	44.5%	60.6%	0.0%	55.0%
**Household income from pensions (controlling also for number of older adults)** Zero inflated negative binomial regression—adjusted^3^ count ratios	Site/ Country	Current care	Incident care	Chronic care	Care exit
	Peru urban	0.92 (0.67–1.27)	0.89 (0.61–1.26)	1.01 (0.62–1.63)	0.88 (0.62–1.24)
	Peru rural	1.04 (0.80–1.36)	1.04 (0.74–1.45)	1.06 (0.77–1.45)	0.93 (0.69–1.24)
	Mexico urban	1.09 (0.70–1.69)	1.15 (0.74–1.80)	0.93 (0.35–2.43)	**1.72 (1.05–2.83)**
	Mexico rural	0.94 (0.71–1.25)	0.90 (0.68–1.20)	1.08 (0.60–1.97)	1.85 (0.62–5.53)
	China urban	1.11 (0.87–1.42)	1.14 (0.87–1.49)	1.05 (0.69–1.59)	0.96 (0.62–1.50)
	China rural	1.19 (0.74–1.92)	1.13 (0.69–1.87)	**2.41 (1.10–5.28)**	**2.24 (1.35–3.73)**
	Pooled CR	1.03 (0.91–1.17)	1.02 (0.89–1.17)	1.10 (0.90–1.34)	1.11 (0.94–1.32)
	I squared	0.0%	0.0%	0.0%	66.2%
**Equivalised household income from private transfers** Zero inflated negative binomial regression—adjusted^3^ count ratios	Site/ Country	Current care	Incident care	Chronic care	Care exit
	Peru urban	0.96 (0.67–1.38)	0.85 (0.55–1.32)	1.18 (0.69–2.02)	1.20 (0.63–2.31)
	Peru rural	0.98 (0.48–2.00)	0.99 (0.48–2.03)	None with income	None with income
	Mexico urban	1.12 (0.91–1.39)	1.16 (0.92–1.47)	0.97 (0.68–1.38)	0.82 (0.58–1.16)
	Mexico rural	0.75 (0.29–1.98)	0.76 (0.30–1.92)	None with income	0.84 (0.19–3.71)
	China urban	1.13 (0.68–1.87)	1.34 (0.80–2.25)	0.54 (0.26–1.14)	0.51 (0.25–1.07)
	China rural	0.59 (0.25–1.39)	0.64 (0.26–1.57)	0.40 (0.04–3.70)	0.38 (0.08–1.70)
	Pooled CR	1.04 (0.89–1.23)	1.07 (0.89–1.28)	0.93 (0.71–1.22)	0.80 (0.61–1.05)
	I squared	0.0%	0.0%	12.3%	0.0%
**Equivalised household income from government transfers** Zero inflated negative binomial regression—adjusted^3^ count ratios	Site/ Country	Current care	Incident care	Chronic care	Care exit
	Peru urban	2.45 (0.81–7.42)	1.18 (0.35–4.07)	None with income	1.32 (0.53–3.33)
	Peru rural	None with income	None with income	None with income	None with income
	Mexico urban	**0.79 (0.67–0.94)**	**0.80 (0.66–0.97)**	0.78 (0.59–1.05)	0.80 (0.61–1.07)
	Mexico rural	1.21 (0.73–2.02)	0.92 (0.30–2.80)	0.47 (0.19–1.16)	0.88 (0.40–1.92)
	China urban	**0.54 (0.35–0.85)**	**0.53 (0.32–0.89)**	0.63 (0.31–1.29)	5.43 (2.58–11.42)
	China rural	0.65 (0.18–2.37)	0.83 (0.26–2.60)	0.13 (0.01–2.74)	0.78 (0.23–2.62)
	Pooled CR	**0.80 (0.69–0.93)**	**0.77 (0.65–0.92)**	**0.72 (0.56–0.93)**	1.01 (0.80–1.28)
	I squared	58.3%	0.0%	0.0%	82.5%

^1^. The reference category in each case is the ‘no care’ group of households, n = 424.

^2^. Equivalised income is total household income adjusted for household size, by dividing by (1 + (0.5 x number of adults beyond 1) + (0.3 x number of children)).

^3^. All estimates are controlled for household assets at baseline, occupational class (highest among older people at baseline), and household composition at baseline (older person alone, with spouse only, with other adults, with other adults and children).

Total household expenditure was lower in chronic care households compared with no care households (CR 0.88, 95% CI 0.77–0.99, I^2^ = 0.0%), but not in incident care households (Table 5). A similar trend was apparent for food consumption. There was a trend towards more indicators of economic strain among care households (CR 1.37, 95% CI 0.97–1.92, I^2^ = 53.3%). Dissatisfaction with economic circumstances was more prevalent in chronic care than in no care households (CR 1.74, 95% CI 1.02–2.97, I^2^ = 66.8%).

**Table 5:** Associations between redesignated household status (no care versus incident care, chronic care and care exit households) and indicators of household consumption, strain and satisfaction.

**Equivalised**^2^ **household consumption** Negative binomial regression—adjusted^3^ count ratios	Site/ Country	Current care^1^ n = 292	Incident care^1^ n = 225	Chronic care^1^ n = 67	Care exit^1^ n = 156
	Peru urban	0.97 (0.84–1.12)	1.03 (0.88–1.21)	0.82 (0.67–1.01)	1.05 (0.88–1.26)
	Peru rural	**0.71 (0.52–0.97)**	**0.67 (0.48–0.94)**	0.96 (0.55–1.68)	1.06 (0.64–1.77)
	Mexico urban	1.06 (0.91–1.24)	1.11 (0.93–1.32)	0.92 (0.72–1.17)	0.95 (0.78–1.16)
	Mexico rural	0.94 (0.77–1.14)	0.91 (0.74–1.12)	1.06 (0.75–1.49)	1.05 (0.83–1.33)
	China urban	0.99 (0.84–1.16)	1.05 (0.88–1.26)	0.78 (0.59–1.03)	0.98 (0.77–1.24)
	China rural	0.76 (0.57–1.03)	**0.76 (0.57–1.00)**	1.03 (0.44–2.44)	1.05 (0.79–1.40)
	Pooled CR	0.96 (0.89–1.03)	0.98 (0.90–1.06)	**0.88 (0.77–0.99)**	1.01 (0.92–1.12)
	I squared	35.6%	56.3%	0%	0%
**Equivalised**^2^ **household food consumption** Negative binomial regression—adjusted^3^ count ratios	Site/ Country	Current care	Incident care	Chronic care	Care exit
	Peru urban	0.92 (0.80–1.06)	0.96 (0.81–1.14)	0.82 (0.66–1.01)	1.07 (0.89–1.29)
	Peru rural	0.91 (0.70–1.19)	0.87 (0.65–1.16)	1.12 (0.69–1.80)	1.20 (0.78–1.85)
	Mexico urban	1.01 (0.83–1.22)	1.11 (0.90–1.38)	0.75 (0.56–1.02)	0.97 (0.76–1.24)
	Mexico rural	1.19 (0.96–1.47)	1.16 (0.92–1.46)	1.38 (0.95–1.99)	1.16 (0.90–1.51)
	China urban	1.01 (0.85–1.21)	1.07 (0.87–1.30)	0.84 (0.62–1.13)	0.97 (0.75–1.25)
	China rural	0.91 (0.63–1.30)	0.84 (0.61–1.17)	2.38 (0.88–6.46)	1.08 (0.77–1.51)
	Pooled CR	0.98 (0.91–1.07)	1.02 (0.93–1.11)	0.90 (0.79–1.03)	1.06 (0.95–1.17)
	I squared	0.0%	0.3%	58.1%	0.0%
**Economic strain indicators—last three years** Ordinal regression—adjusted^3^ odds ratios	Site/ Country	Current care	Incident care	Chronic care	Care exit
	Peru urban	**2.46 (1.15–5.27)**	1.85 (0.79–4.38)	**3.70 (1.28–10.72)**	2.30 (0.84–6.30)
	Peru rural	3.18 (0.86–11.68)	**4.14 (1.08–15.85)**	0.88 (0.10–7.95)	2.83 (0.42–19.32)
	Mexico urban	0.72 (0.39–1.36)	0.73 (0.37–1.48)	0.64 (0.23–1.80)	0.66 (0.31–1.40)
	Mexico rural	1.43 (0.73–2.80)	1.66 (0.82–3.39)	0.83 (0.23–3.04)	1.62 (0.70–3.74)
	China urban	2.59 (0.86–7.86)	2.17 (0.62–7.57)	3.91 (0.89–17.19)	0.77 (0.08–6.95)
	China rural	0.63 (0.18–2.20)	0.51 (0.14–1.90)	16.86 (0.50–566.55)	0.69 (0.21–2.28)
	Pooled OR	1.37 (0.97–1.92)	1.33 (0.92–1.94)	1.58 (0.90–2.77)	1.15 (0.75–1.77)
	I squared	53.3%	45.8%	49.2%	19.7%
**Dissatisfaction with economic circumstances** Ordinal regression—adjusted^3^ odds ratios	Site/ Country	Current care	Incident care	Chronic care	Care exit
	Peru urban	1.48 (0.68–3.24)	1.09 (0.45–2.62)	2.02 (0.66–6.13)	1.20 (0.44–3.28)
	Peru rural	**4.19 (1.18–14.85)**	**6.89 (1.77–26.81)**	1.00 (0.13–7.74)	**7.05 (1.00–49.62)**
	Mexico urban	0.91 (0.49–1.69)	0.96 (0.48–1.93)	0.89 (0.34–2.28)	0.73 (0.35–1.51)
	Mexico rural	0.78 (0.37–1.63)	0.85 (0.40–1.83)	0.72 (0.22–2.39)	**0.37 (0.15–0.93)**
	China urban	**2.23 (1.10–4.49)**	1.29 (0.58–2.80)	**13.79 (3.80–50.01)**	0.95 (0.33–2.74)
	China rural	0.81 (0.24–2.74)	0.57 (0.17–1.94)	8.12 (0.27–247.58)	0.38 (0.10–1.42)
	Pooled OR	1.28 (0.92–1.77)	1.11 (0.78–1.58)	**1.74 (1.02–2.97)**	0.70 (0.46–1.06)
	I squared	46.3%	43.6%	66.8%	57.7%

^1^. The reference category in each case is the ‘no care’ group of households, n = 424.

^2^. Equivalised consumption is total household consumption adjusted for household size, by dividing by (1 + (0.5 x number of adults beyond 1) + (0.3 x number of children)).

^3^. All estimates are controlled for household assets at baseline, occupational class (highest among older people at baseline), and household composition at baseline (older person alone, with spouse only, with other adults, with other adults and children).

Healthcare costs were significantly higher among care households in some sites (particularly the urban sites in Peru and China), and in the pooled estimate (CR 1.55, 95% CI 1.26–1.90, I^2^ = 73.4%) but with considerable heterogeneity among sites (Table 6). Catastrophic healthcare spending was significantly more likely in care households (PR 1.64, 95% CI 1.20–2.22, I^2^ = 16.3%), with much less heterogeneity of effect between sites. The striking finding in this section of the analysis was that while elevated household healthcare spending was apparent across incident and chronic care households, household healthcare costs in care exit households were significantly lower than in no care households. Compared with no care households, not working, or giving up education to care for an older household member was considerably more common in both incident care (PR 2.08, 95% CI 1.37–3.16, I^2^ = 32.3%) and chronic care households (PR 2.22, 95% CI 1.43–3.43, I^2^ = 48.3%).

**Table 6:** Associations between household status (no care vs incident care, chronic care and care exit) and out of pocket healthcare expenditure, catastrophic healthcare spending, and not engaging in education or paid work to care for an older adult.

**Household healthcare expenditure** Zero inflated negative binomial regression—adjusted^1^ count ratios		Current care^1^ n = 292	Incident^1^ n = 225	Chronic^1^ n = 67	Care exit^1^ n = 156
	Peru urban	**2.32 (1.68–3.20)**	**2.08 (1.46–2.97)**	**2.14 (1.43–3.20)**	0.68 (0.42–1.11)
	Peru rural	0.79 (0.27–2.35)	**0.46 (0.25–0.84)**	1.21 (0.45–3.28)	**0.07 (0.03–0.17)**
	Mexico urban	0.86 (0.50–1.47)	0.94 (0.51–1.73)	0.64 (0.27–1.51)	0.68 (0.30–1.51)
	Mexico rural	0.79 (0.46–1.37)	0.63 (0.35–1.12)	1.39 (0.52–3.76)	0.48 (0.19–1.18)
	China urban	**1.87 (1.23–2.83)**	**1.99 (1.27–3.11)**	0.68 (0.33–1.41)	1.39 (0.67–2.87)
	China rural	1.37 (0.52–3.59)	2.19 (0.72–6.61)	0.78 (0.05–13.50)	**0.06 (0.02–0.16)**
	Pooled CR	**1.55 (1.26–1.90)**	**1.33 (1.07–1.64)**	**1.40 (1.04–1.87)**	**0.46 (0.34–0.62)**
	I squared	73.4%	83.0%	56.5%	88.9%
**Catastrophic healthcare expenditure** Poisson regression—adjusted^2^ relative risks		Current care	Incident care	Chronic care	Care exit
	Peru urban	**4.88 (1.73–13.79)**	**4.48 (1.46–13.73)**	**5.67 (1.66–19.34)**	None with outcome
	Peru rural	2.36 (0.53–10.63)	2.34 (0.48–11.34)	11.29 (0.30–419.71)	None with outcome
	Mexico urban	1.55 (0.85–2.85)	1.72 (0.90–3.27)	1.17 (0.44–3.14)	0.46 (0.13–1.58)
	Mexico rural	1.14 (0.61–2.15)	1.28 (0.66–2.49)	0.75 (0.22–2.60)	0.60 (0.20–1.77)
	China urban	1.70 (0.94–3.11)	1.79 (0.95–3.40)	1.44 (0.52–3.99)	1.06 (0.42–2.64)
	China rural	1.36 (0.57–3.27)	1.30 (0.53–3.19)	2.21 (0.38–17.61)	0.14 (0.02–1.08)
	Pooled PR	**1.64 (1.20–2.22)**	**1.71 (1.24–2.37)**	1.67 (0.99–2.81)	0.63 (0.35–1.13)
	I squared	16.3%	0%	30.2%	18.2%
**Another resident is not working (main reason cited is to care for older person)** Poisson regression—adjusted^3^ relative risks		Current care	Incident care	Chronic care	Care exit
	Peru urban	2.05 (0.85–4.94)	2.31 (0.90–5.92)	1.53 (0.41–5.70)	Omitted
	Peru rural	**5.98 (1.36–26.18)**	**7.01 (1.52–32.45)**	2.15 (0.15–30.61)	Omitted
	Mexico urban	**5.12 (1.87–14.01)**	**5.50 (1.94–15.60)**	**4.18 (1.11–15.73)**	Omitted
	Mexico rural	1.35 (0.52–3.49)	1.38 (0.50–3.79)	1.27 (0.25–6.40)	Omitted
	China urban	0.90 (0.04–18.91)	None exposed	11.65 (0.02–5699.85)	Omitted
	China rural	1.36 (0.65–2.84)	1.33 (0.63–2.81)	1.95 (0.25–15.20)	Omitted
	Pooled PR	**2.08 (1.37–3.16)**	**2.22 (1.43–3.43)**	**2.15 (1.04–4.42)**	Omitted
	I squared	32.3%	48.3%	0%	

^1^. The reference category in each case is the ‘no care’ group of households, n = 424.

^2^. Controlled for household assets at baseline, occupational class (highest among older people at baseline), and household composition at baseline (older person alone, with spouse only, with other adults, with other adults and children), and number of adult and number of child residents.

^3^. Controlled for household assets at baseline, occupational class (highest among older people at baseline), and household composition at baseline (older person alone, with spouse only, with other adults, with other adults and children).

As a sensitivity analysis, we limited the analysis to those households that were stable from the incidence wave (hence excluding the effect of household changes), and also excluded no care households where older residents were found to have developed needs for care (S1 Table). Patterns of association, and effect sizes were generally similar to those from the main analyses. However, when comparing care households with no care households, the effects of care dependence on economic strain (OR 1.64, 95% CI 1.11–2.44, I^2^ = 60.6%) and dissatisfaction with economic circumstances (OR 1.50, 95% CI 1.04–2.18, I^2^ = 60.6%) were clarified.

## Discussion

### Principal findings

In our cohort study in rural and urban catchment area sites in Peru, Mexico and China, we classified households according to the needs for care of older residents across two waves of population surveys. At the second of these waves there were no differences in household assets between households defined as providing ‘no care’, ‘incident care’ or ‘chronic care’. However, when followed up three years later for this nested INDEP cohort study we found that while total household incomes were similar between groups, income from paid work and government transfers was lower in care than in no care households, expenditure on healthcare was higher, and catastrophic healthcare expenditure more common. Consumption was lower in chronic care than no care households, but similar in incident care households.

### Strengths and weaknesses of the study

The main strength of our study is its longitudinal perspective. Household exposure to older adult care dependence was established in the 10/66 baseline and incidence wave surveys over a three to five year period, ending three years before the assessment of economic outcomes in the nested INDEP study. Household assets, and socioeconomic position based upon the highest occupational class among the residents (both indicators of long-term household economic status) were similar among household categories for the households selected into the INDEP study, and these variables, as assessed at the incidence wave of the 10/66 survey, were also controlled for in all models. Unfortunately these earlier 10/66 surveys did not include measures of household income and consumption, which would have provided further control for the effects of earlier economic disadvantage on care dependence. Importantly, all INDEP study outcomes, including health and social care costs were assessed at household level, and not only for the IOP. Excessive care demands are known to have an adverse effect on caregiver health [30].

The rich description of the health circumstances of the IOP from the previous two waves of 10/66 surveys allowed us to validate and contextualise the classification of no care, incident care and chronic care households, with a high prevalence of dementia and stroke among IOP in chronic care households at both time points, and in incident care households at the incidence wave, and a rising mean disability score in incident care households [24]. At the incidence wave survey, dementia affected up to half of IOP in the incident care households, and two-thirds in the chronic care households, underlining the typically chronic and progressive nature of needs for care. Health conditions and disability give rise to the needs for care that may lead to economic adversity, and are therefore not controlled for in the analyses presented here.

The main limitations of the study are the catchment area sampling, the changes of residence and health status, and attrition. The catchment area sampling limits generalisability, since the catchment areas, although carefully characterised, may not be representative of urban or rural settings in general in the countries concerned. Attrition arose for several reasons. First, deaths of care dependent older people lead to redesignation of incident and chronic care households as ‘care exit’. This reduced the numbers of incident and, particularly, chronic care households, but did allow us to assess the economic status of such households after care demands had ceased. A smaller number of no care households were lost because of the deaths of all IOP. Second, only 68% of eligible households could be traced and interviewed, much of the non-response occurring in the ‘care exit’ households. Other than in this group, refusal was rare, and most non-response arose from difficulties in tracing the relevant IOP to the household where they now resided, particularly in the urban China catchment area, due to extensive redevelopment and compulsory displacement around the time of the Beijing Olympics. The losses to follow up in China are most likely to have been non-differential with respect to exposure and outcomes, and therefore unlikely to have resulted in bias. Those in other sites may have biased estimates in either direction if moving away was linked, as it may have been, to both needs for care, and household economic functioning. The weighting of the analysis for non-response as well as sampling may have partly compensated for this problem. The reallocation of care households to the care exit group, coupled with losses to follow-up reduced the power and precision of the analyses that we conducted, as indicated by the breadth of the 95% confidence intervals. This may mean that some analyses were relatively underpowered, particularly at the level of individual sites. This was unavoidable given that sample size and power were limited by the availability of care households at the incidence wave, all of which were selected for the INDEP study.

Overall 8.9% of households were subject to household change, due to relocation of IOP. This applied to 35.1% of households in rural China, and 13.7% in rural Mexico, and was relatively uncommon in other sites. For the most part the household changes resulted from the IOP moving into another household, rather than the whole household moving en masse to another location. This was a common theme in the qualitative research, particularly for rural China, where older people with needs for care sometimes rotated among their children’s homes to share care demands and costs, or moved to another household in the extended family network where those demands could be more conveniently and adequately met [25]. These household changes, and changes in household composition when incoming residents bring additional income or capacity to care, are informal mechanisms that mitigate the economic and social impact of older adult care dependence [25]. It is difficult to decide how they should best be addressed. We conducted a sensitivity analysis, in which, following the exclusion of households subject to household changes, the effect sizes for most of the associations with economic disadvantage or strain increased in magnitude. This issue seems to have been little considered previously, probably because of the preponderance of cross-sectional studies.

### Inferences and mechanisms

The main challenge to interpretation of our findings is that of endogeneity; that is that pre-existing socioeconomic disadvantage could have been a determinant of the development of care dependence, while also explaining the observed decrements in household consumption and components of household income in care households (uncontrolled confounding/ reverse causality), and/ or that economic disadvantage and poor health covary across the life course (simultaneity). Control for household assets and occupational level partly addresses these concerns, but earlier measures of household income and consumption would have helped to clarify causality, and its direction. Nevertheless, our findings, taken together, support a pathway from late-life care dependence to household economic disadvantage and adversity, while not excluding an association in the reverse direction. Household expenditure on health and social care was substantially lower in care exit households than no care households, suggesting generally elevated costs in households with older residents. However, among these households, expenditure was concentrated among care households. Associations with lower consumption, economic strain and dissatisfaction with economic circumstances were all more prominent for chronic care households than the more recently incident care households, suggesting a possible cumulative effect of care dependence over time. The long-term increased out-of-pocket costs of health and social care provide one likely explanation for the lower consumption in chronic care households, despite similar total household incomes. Restricting consumption of food and other household expenditure was a commonly strategy for care households across countries in our accompanying qualitative research, often provoked by falling into debt [25]. Income from paid work was lower in care households, probably accounted for by work incapacity in the IOP, as well as working age adult residents forgoing working opportunities to care. Our qualitative research also highlighted that caregivers were often limited to part-time flexible work that was less well paid than previous jobs, and for which they were over-qualified [25]. These losses were evidently not compensated by income from government transfers, which were also significantly lower in care households. Private transfers of cash or goods from outside of the home did little to decrease the economic impact of care dependence, since these were distributed equally between care and no care households. In our qualitative research, the unpredictability and unreliability of private transfers limited their practical value to beneficiaries [25]. The non-significantly higher income from pensions in care households in some sites, and the much higher income from assets in urban Peru and urban China probably account for the overall null effect of care dependence on total household income. Monetisation of assets (representing a deterioration in household economic security, even if temporarily effective in maintaining household income) was another important coping mechanism identified in our qualitative research [25].

### Contextualisation with other research

The impoverishing effects of older adult care dependence are widely discussed, but little studied in LMIC [18]. Social and economic protection for older people is much more limited than in HIC, with low pension coverage, and a high reliance on out-of-pocket payments for healthcare. There are, furthermore, no structured systems of social care to support, supplement or substitute for family informal care, if they lack capacity to meet the demand [25]. Government policy (for example introduction of social pensions, conditional cash transfers, and health insurance), and economic development have led to some recent improvements for older people in the INDEP study countries, but gross inequalities persist determined mainly by the limited reach of the formal labour market and the access it brings to government contributory pension and health insurance schemes [31].

Our findings are broadly consistent with those from previous studies in HIC, in suggesting an association between disability in older adult residents and household economic disadvantage and strain. Our headline finding of a 12% lower consumption level in chronic care households (based upon count ratios meta-analysed across sites, with no heterogeneity), controlling for baseline household socioeconomic status and household composition, is similar to the cost of disability in Vietnam, amounting to around 9% of household income using the ‘standard of living’ approach [19]. This, and other studies have emphasized that state benefits fail to compensate for the increased costs of disability and needs for care, even in states with relatively well developed welfare systems [12–17]. Nevertheless, such inequities could be reduced through social pensions, poverty alleviation cash transfers (e.g. ‘70 y Mas’ in Mexico [31,32]), and more targeted benefits in the form of caregiver allowances and disability pensions. Caregiver allowances do not exist in the countries studied, and disability benefits have very minimal coverage. The targeting and implementation of health insurance schemes need to be carefully thought through, if they are to meet the needs of older people. Enrolment can be patchy and inequitable [33,34] and insurance schemes often do not meet costs associated with chronic disease (outpatient care, medication, transport, dressings) that account for the majority of out-of-pocket costs, particularly for older adults [25,34,35].

## Conclusions

In summary, we report some of the first direct and detailed evidence, from middle income countries of a discernable negative economic impact on household-level economic functioning associated with care dependence of older adult residents. While this is a longitudinal study with careful control for previous household economic status we cannot confidently attribute causality, since there may have been some covariance of economic and health disadvantage over the life-course, and/ or uncontrolled confounding given the lack of baseline measures of household income and consumption. Nevertheless, given the associated high direct costs of health and social care, and livelihood opportunity costs of caregivers, the associations are highly plausible. The elucidation of these effects at household level is an important finding. The needs of older people have, hitherto, never been prominent in the global health and development agendas. This study emphasizes that the health and wellbeing of older people, living, typically, in multigenerational households, and largely dependent upon their families for their basic needs, is inextricably linked with that of the household unit and extended family [36]. Population ageing will rapidly increase the numbers of ‘households where older people live’ and their societal significance. Numbers of care dependent older people may quadruple in LMIC through to 2050, while numbers of younger care dependent people remain stable [37]. An urgent policy response is needed to make long-term care arrangements sustainable into the future. This is likely to require some combination of improved income security in old age (social pensions, and greater access to contributory schemes), incentivisation of informal care through compensation for direct and opportunity costs (disability benefits and caregiver allowances), and incremental provision of structured social care services to support, and, where necessary, supplement or substitute the central role of informal caregivers.

## Supporting information

S1 Table Sensitivity analysis (excluding household changes, and no care households where one or more older residents have developed needs for care). Associations between redesignated household status (no care versus incident care, chronic care and care exit households) and main household economic welfare indicators (income, consumption, strain and satisfaction).
(DOCX)
