# Supplementary material for: A cohort study of the effects of older adult care dependence upon household economic functioning, in Peru, Mexico and China
Source: PLoS One. 2018 Apr 13;13(4):e0195567. doi: 10.1371/journal.pone.0195567 (PMC5898721; doi:10.1371/journal.pone.0195567)
Supplement: S1 Table — Associations between redesignated household status (no care versus incident care, chronic care and care exit households) and main household economic welfare indicators (income, consumption, strain and satisfaction). (DOCX) [file pone.0195567.s001.docx]

S1 Table

Sensitivity analysis (excluding household changes, and no care households where one or more older residents have developed needs for care). Associations between redesignated household status (no care versus incident care, chronic care and care exit households) and main household economic welfare indicators (income, consumption, strain and satisfaction)

| **Equivalised Household income**  Negative binomial regression – adjusted count ratios | Site/ Country | No care  n=260 | All care  n=254 | Incident  n=192 | Chronic  n=62 | Care exit  n=91 |
| --- | --- | --- | --- | --- | --- | --- |
|  | Peru urban | 1 (ref) | 1.07 (0.91-1.27) | 1.06 (0.88-1.28) | 1.09 (0.86-1.38) | 1.05 (0.85-1.29) |
|  | Peru rural | 1 (ref) | 0.83 (0.55-1.25) | 0.81 (0.52-1.28) | 1.10 (0.49-2.48) | 1.00 (0.52-1.88) |
|  | Mexico urban | 1 (ref) | 1.01 (0.81-1.26) | 1.04 (0.82-1.33) | 0.87 (0.62-1.22) | 1.11 (0.78-1.58) |
|  | Mexico rural | 1 (ref) | 0.97 (0.72-1.31) | 1.02 (0.73-1.41) | 0.86 (0.51-1.45) | 1.09 (0.62-1.92) |
|  | China urban | 1 (ref) | **1.62 (1.16-2.26)** | **1.84 (1.30-2.60)** | 1.09 (0.67-1.75) | 0.73 (0.48-1.12) |
|  | China rural | 1 (ref) | 0.49 (0.24-1.02) | 0.60 (0.28-1.28) | 0.22 (0.02-2.02) | 1.94 (0.84-4.46) |
|  | Pooled CR | 1 (ref) | 1.05 (0.94-1.17) | **1.20 (1.09-1.33)** | 1.00 (0.85-1.18) | 1.03 (0.89-1.20) |
|  | I squared |  | 59.9% | 88.4% | 0.0% | 0.0% |
| **Equivalised Household expenditure**  Negative binomial regression – adjusted count ratios | Site/ Country | No care | All care | Incident | Chronic | Care exit |
|  | Peru urban | 1 (ref) | 0.96 (0.82-1.12) | 1.01 (0.85-1.21) | 0.82 (0.66-1.03) | 1.06 (0.88-1.29) |
|  | Peru rural | 1 (ref) | **0.65 (0.47-0.89)** | **0.61 (0.48-0.87)** | 0.89 (0.48-1.66) | 1.00 (0.60-1.68) |
|  | Mexico urban | 1 (ref) | 1.09 (0.92-1.29) | 1.14 (0.95-1.37) | 0.94 (0.73-1.22) | 1.06 (0.82-1.37) |
|  | Mexico rural | 1 (ref) | 0.98 (0.79-1.21) | 0.96 (0.77-1.21) | 1.04 (0.72-1.48) | 1.05 (0.71-1.55) |
|  | China urban | 1 (ref) | 0.94 (0.78-1.12) | 1.00 (0.82-1.22) | **0.74 (0.55-0.99)** | 0.93 (0.72-1.19) |
|  | China rural | 1 (ref) | **0.47 (0.31-0.72)** | **0.51 (0.34-0.75)** | 0.43 (0.13-1.37) | 1.05 (0.79-1.40) |
|  | Pooled CR | 1 (ref) | 0.94 (0.86-1.02) | 0.95 (0.87-1.04) | **0.86 (0.75-0.98)** | 1.03 (0.92-1.15) |
|  | I squared |  | 73.2% | 77.8% | 0.0% | 0.0% |
| **Equivalised food consumption**  Negative binomial regression – adjusted count ratios | Site/ Country | No care | All care | Incident | Chronic | Care exit |
|  | Peru urban | 1 (ref) | 0.89 (0.77-1.03) | 0.92 (0.77-1.09) | 0.83 (0.66-1.03) | 1.05 (0.87-1.28) |
|  | Peru rural | 1 (ref) | 0.88 (0.66-1.18) | 0.84 (0.62-1.15) | 1.12 (0.66-1.91) | 1.17 (0.75-1.82) |
|  | Mexico urban | 1 (ref) | 1.04 (0.84-1.28) | 1.14 (0.90-1.43) | 0.78 (0.57-1.06) | 1.00 (0.73-1.37) |
|  | Mexico rural | 1 (ref) | 1.26 (0.99-1.60) | 1.25 (0.97-1.62) | 1.29 (0.87-1.93) | 0.99 (0.63-1.57) |
|  | China urban | 1 (ref) | 1.02 (0.83-1.24) | 1.07 (0.87-1.32) | 0.86 (0.63-1.17) | 0.97 (0.74-1.26) |
|  | China rural | 1 (ref) | 0.70 (0.41-1.19) | 0.66 (0.41-1.08) | 1.88 (0.45-7.92) | 1.09 (0.62-1.91) |
|  | Pooled CR | 1 (ref) | 0.98 (0.90-1.07) | 1.01 (0.92-1.11) | 0.90 (0.78-1.03) | 1.03 (0.91-1.17) |
|  | I squared |  | 40.6% | 47.0% | 19.5% | 0.0% |
| **Strain in the past three years**  Ordinal regression – adjusted odds ratios | Site/ Country | No care | All care | Incident | Chronic | Care exit |
|  | Peru urban | 1 (ref) | 2.14 (0.93-4.89) | 1.42 (0.56-3.60) | **3.90 (1.25-12.2)** | 2.29 (0.79-6.68) |
|  | Peru rural | 1 (ref) | 3.32 (0.83-13.3) | **4.28 (1.03-17.7)** | 0.67 (0.06-8.00) | 2.90 (0.41-20.4) |
|  | Mexico urban | 1 (ref) | 0.95 (0.48-1.89) | 0.93 (0.44-1.98) | 0.95 (0.33-2.76) | 0.99 (0.34-2.82) |
|  | Mexico rural | 1 (ref) | **2.66 (1.20-5.91)** | **3.10 (1.33-7.20)** | 1.61 (0.40-6.43) | 1.56 (0.98-7.01) |
|  | China urban | 1 (ref) | 4.64 (0.93-23.06) | 3.34 (0.59-18.76) | **9.08 (1.36-60.54)** | 1.51 (0.13-18.0) |
|  | China rural | 1 (ref) | 0.21 (0.04-1.12) | **0.15 (0.02-0.91)** | 4.36 (0.10-191.7) | 0.43 (0.05-4.83) |
|  | Pooled CR | 1 (ref) | **1.64 (1.11-2.44)** | **1.58 (1.03-2.42)** | **2.03 (1.10-3.73)** | 1.49 (0.87-2.56) |
|  | I squared |  | 60.6% | 61.1% | 25.1% | 0.0% |
| **Satisfaction with economic circumstances**  Ordinal regression – adjusted odds ratios | Site/ Country | No care |  |  |  |  |
|  | Peru urban | 1 (ref) | 1.83 (0.78-4.34) | 1.23 (0.48-3.13) | 2.60 (0.80-8.43) | 1.46 (0.51-4.22) |
|  | Peru rural | 1 (ref) | 3.14 (0.81-12.06) | 6.26 (1.49-26.32) | 0.65 (0.07-6.17) | 5.88 (0.83-41.67) |
|  | Mexico urban | 1 (ref) | 0.91 (0.46-1.80) | 0.95 (0.45-1.99) | 1.07 (0.40-2.87) | 0.73 (0.26-2.01) |
|  | Mexico rural | 1 (ref) | 0.93 (0.40-2.13) | 1.06 (0.43-2.58) | 0.64 (0.17-2.49) | 0.10 (0.02-0.52) |
|  | China urban | 1 (ref) | **4.38 (1.81-10.57)** | 2.54 (0.98-6.58) | 28.05 (6.87-114.44) | 1.99 (0.62-6.46) |
|  | China rural | 1 (ref) | 0.78 (0.17-3.69) | 0.68 (0.13-3.41) | DNC | 0.87 (0.61-52.90) |
|  | Pooled CR | 1 (ref) | **1.50 (1.04-2.18)** | 1.38 (0.92-2.07) | **2.02 (1.13-3.59)** | 1.03 (0.60-1.77) |
|  | I squared |  | 54.7% | 36.9% | 79.0% | 61.5% |

Notes

1. All estimates are controlled for household assets at baseline, occupational class (highest among older people at baseline), and household composition at baseline (older person alone, with spouse only, with other adults, with other adults and children)

2. Equivalised income and consumption is total household income or consumption adjusted for household size, by dividing by (1 + (0.5 x number of adults beyond 1) + (0.3 x number of children))
